# Mechanistic insights into connexin-mediated neuroglia crosstalk in neurodegenerative diseases

**DOI:** 10.3389/fncel.2025.1532960

**Published:** 2025-02-11

**Authors:** Simona Denaro, Simona D’Aprile, Nunzio Vicario, Rosalba Parenti

**Affiliations:** Section of Physiology, Department of Biomedical and Biotechnological Sciences, University of Catania, Catania, Italy

**Keywords:** chronic neuroinflammation, neurodegeneration, connexin 43, Alzheimer’s disease, Parkinson’s disease, multiple sclerosis, Huntington’s disease

## Abstract

Neurodegenerative diseases, such as Alzheimer’s disease (AD), Parkinson’s disease (PD), Multiple Sclerosis (MS), and Huntington’s disease (HD), although distinct in their clinical manifestations, share a common hallmark: a disrupted neuroinflammatory environment orchestrated by dysregulation of neuroglial intercellular communication. Neuroglial crosstalk is physiologically ensured by extracellular mediators and by the activity of connexins (Cxs), the forming proteins of gap junctions (Gjs) and hemichannels (HCs), which maintain intracellular and extracellular homeostasis. However, accumulating evidence suggests that Cxs can also act as pathological pore in neuroinflammatory conditions, thereby contributing to neurodegenerative phenomena such as synaptic dysfunction, oxidative stress, and ultimately cell death. This review explores mechanistic insights of Cxs-mediated intercellular communication in the progression of neurodegenerative diseases and discusses the therapeutic potential of targeting Cxs to restore cellular homeostasis.

## 1 Introduction

Neurodegenerative diseases are characterized by progressive neuronal loss in the central nervous system (CNS) and/or peripheral nervous system (PNS). To date, standard therapies for neurodegenerative diseases are not curative, and patient improvements are limited, highlighting the need for more effective treatments (Dejanovic et al., 2024). Neurodegenerative diseases, such as Alzheimer’s disease (AD), Parkinson’s disease (PD), multiple sclerosis (MS) and Huntington’s disease (HD), represent a diverse group of disorders with distinct clinical and pathological features (Erkkinen et al., 2018). The heterogeneity of these disorders is based on clinical symptoms such as cognitive decline and motor impairments and areas affected by neurodegeneration, ranging from brain regions critical for memory and movement to the myelin sheath in MS (Dugger and Dickson, 2017; Giallongo et al., 2022).

A shared hallmark between these diseases is the accumulation of proteins aggregates, such as the amyloid-beta (Aβ) plaques or misfolded protein and neuroinflammatory mediators, which drive oxidative stress, neurodegeneration and cell death (Huang et al., 2021; Giallongo et al., 2022). Neuroinflammation is strongly supported by resident glial cells activation, including microglia and astrocytes, which modulate neuronal activity leading to alterations in their function and morphology. The CNS is an immune privileged tissue, and microglia represent the resident immune cells acting as phagocyting cells and protecting CNS and contributing to homeostasis (Muzio et al., 2021). Solid evidence suggest that their functions and phenotype can be associated with neuroinflammatory and neurotoxic stimulation and it has been linked to CNS degenerative diseases (Muzio et al., 2021). Reactive astrocytes may also contribute to neurodegeneration and show synaptotoxic effects, contributing to synapse alterations and neuronal suffering in preclinical models (Dugger and Dickson, 2017; Goshi et al., 2020; Kwon and Koh, 2020; Teleanu et al., 2022). In line with this evidence, it has been observed that A1-like astrocytes are abundant in key CNS areas of AD, PD, MS and HD patients, actively driving neuronal death and synaptic loss processes (Liddelow et al., 2017).

**FIGURE 1:**
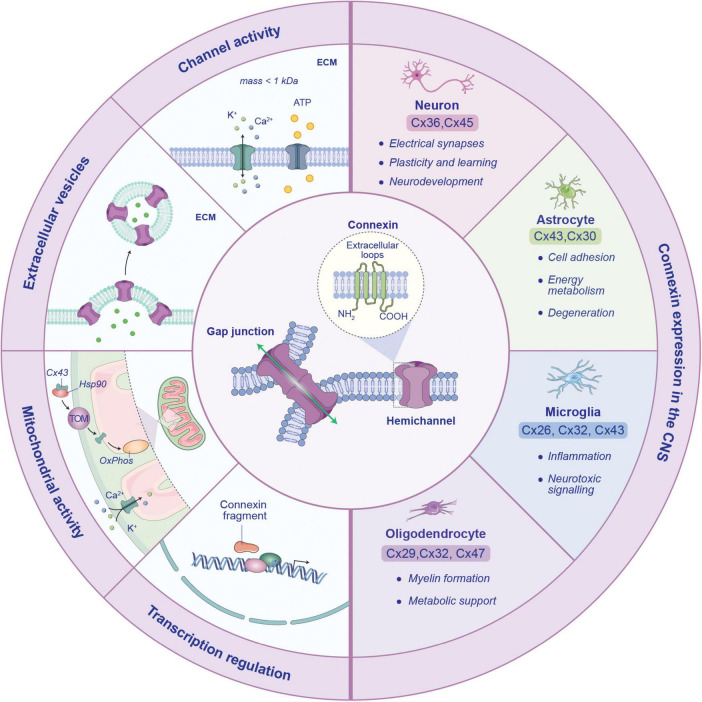
Structure, functions and distribution of connexin isoforms in the central nervous system. Connexins (Cxs) form both hemichannels (HCs) and gap junctions (GJs), facilitating the exchange of ions and small molecules between cells or with the extracellular environment (*channel activity*). Beyond intercellular communication, Cxs also influence *mitochondrial activity*, where they presumably form HCs. Particularly, Cx43 is imported within the inner mitochondria membrane through a pathway involving heat shock protein 90 (Hsp90) and translocate of the outer membrane (TOM) proteins, where it impacts oxidative phosphorylation (OxPhos) and ion homeostasis (Boengler et al., 2022). However, the exact role of mitochondrial Cx in this process is still unclear. Additionally, Cxs are involved in *transcription regulation* and the release of *extracellular vesicles*, extending their influence on central nervous system (CNS) physiology. Different CNS cell types express distinct connexin isoforms. Neuronal Cxs are involved in synaptic plasticity, learning and neurodevelopment, while astrocyte Cxs participate in cell adhesion, energy metabolism and neurodegeneration. Microglia Cxs regulate inflammation and neurotoxic signaling. In oligodendrocytes, Cxs contribute to myelin formation and metabolic support.

Glial cells communicate with each other and with neurons through gap junctions (GJs), which allow to exchange ions and small molecules, such as glucose, glutathione, glutamate, between their intracellular compartments cyclic adenosine monophosphate (cAMP) (Giaume et al., 2021). The structure of GJs is composed by connexins (Cxs), a protein family with four transmembrane domains, two extracellular loops, one intracellular loop and one intracellular carboxy-tail (Vicario and Parenti, 2022). Six Cx units form a hemichannel (HC) and two docked HCs of adjacent cells constitute a GJ. HCs are also involved in the interaction between intracellular and extracellular spaces and can be classified as homomeric or heteromeric, based on their Cxs composition (Vicario and Parenti, 2022; Figure 1). Up to now, 21 human Cx isoforms have been identified and the protein names have been assigned on their molecular weight in kilodaltons (Kutova et al., 2023). In addition to their canonical channel function, Cxs are involved in additional biological processes, including cell migration and metabolism, interaction with cytoskeleton, and mRNA transcription regulation, which collectively may have an indirect impact on intercellular crosstalk (Lucaciu et al., 2023; Su et al., 2024; Figure 1).

Connexins exhibit distinct subtype-specific expression patterns in CNS-resident cells. Neurons primarily express Cx36 and Cx45, mainly involved in electrical synapses formation and synaptic plasticity phenomena. In astrocytes, Cx43 and Cx30 are the predominant isoforms, contributing to cell adhesion, energy metabolism and processes related to neurodegeneration. Microglia express Cx26, Cx32 and Cx43, which are involved in regulating inflammation and neurotoxic signaling. Cx29, Cx32 and Cx47 are predominantly expressed by oligodendrocytes, providing metabolic support and contributing to myelin formation (Vicario et al., 2017; Figure 1).

In the last years, many studies have focused on GJ- and HC-forming Cxs dysregulation that, affecting homeostatic balance and cell membrane permeability, sustains the onset and progression of neurodegenerative diseases (Orellana et al., 2010; Vicario et al., 2017; Jiang et al., 2023). In particular, Cxs expression levels have been associated with several physiological and pathological functions in the CNS, such as memory and behavior, neuroglia homeostasis and reactive gliosis, implying Cxs as new potential targets for the treatment of neurodegenerative diseases (Giaume et al., 2021). Cxs phosphorylation also represents a critical aspect of Cx function, triggered by different kinases in response to intracellular signals, including changes in calcium levels, oxidative stress and activation of signaling pathway. These include the gating (opening and closing) of GJs, the trafficking and assembly of Cxs into the plasma membrane and their degradation. In the context of neurodegenerative processes, altered phosphorylation can disrupt intercellular communication, contributing to pathological remodeling of GJs. However, several aspects remain controversial and need to be further explored (Schulz et al., 2015; Guo et al., 2022).

This review focuses on the involvement of neuroglia in neuroinflammation, exploring how glial Cxs contribute to the pathophysiology and progression of AD, PD, MS, and HD and exploring possibilities to target Cxs as a potential effective strategy for the treatment of neurodegenerative diseases.

## 2 Connexins’ role in Alzheimer’s disease (AD)

Alzheimer’s disease is the most common cause of dementia, characterized by cognitive decline, with pathological features that remain untreatable. Alterations in glial Cxs are implicated in AD neurotoxicity and increased HCs activity exacerbates neurodegeneration and gliotransmitter release (Table 1). Key pathological hallmarks, such as extracellular Aβ plaques and hyperphosphorylated tau aggregates, drive neurodegeneration and synaptic loss (Scheltens et al., 2016; Scheltens et al., 2021). Despite extensive research and numerous therapeutic strategies, no effective treatments are currently available, with prevention remaining the primary approach (Soria Lopez et al., 2019; Passeri et al., 2022). Recent research findings revealed that, beyond neurons, also microglia and astrocytes are involved in AD pathogenesis, showing their role as potential therapeutic targets. Indeed, glial cells sustain neuroinflammatory responses, whose long-term effects are neuronal suffering and cell death (Cragnolini et al., 2020). While microglia activation may result in synapses loss through a complement-dependent process and the secretion of inflammatory mediators (Swanson et al., 2020; McFarland and Chakrabarty, 2022), astrocytes exhibit neurotoxic reactivity triggered by Aβ aggregates and cytokines release (Shao et al., 1997; Stevenson et al., 2020). However, glial-induced inflammation can have both beneficial and detrimental effects in AD (Uddin and Lim, 2022).

**TABLE 1:** Connexins (Cxs) modulation in neurodegenerative diseases.

Disease	Model	Cxs modulation	Effects	References
Alzheimer’s disease	Mouse primary astrocytes treated with Aβ_25–35_	Increased Cx43 intracellular pool and activity	Neurotoxicity and neuronal damage	Maulik et al., 2020
	APPswe/PS1dE9 mouse model	Increased Cx43 HCs activity	High Ca^2+^ levels in astrocytes; neuronal suffering	Yi et al., 2016
	Old 5XFAD mouse model	Increased Cx43 and Cx30, reduced Cx47 expression	Loss of interaction between astrocytes and oligodendrocytes	Angeli et al., 2020
	APP/PS1 mouse model of early AD	Increased Cx43 phosphorylation levels	Unknown	Madeira et al., 2023
	Mouse primary microglia treated with Aβ_25–35_	Increased Cx43 and Panx1 activities	Neurotoxic alterations	Orellana et al., 2012
Parkinson’s disease	Rotenone-induced rat PD model	Increased Cx43 expression and phosphorylation levels	Unknown	Kawasaki et al., 2009
	Rotenone-stimulated primary cultured astrocytes	Reduced Cx43 expression	Dysfunction of astrocyte GJs	Zhang et al., 2011
	Mouse primary astrocytes treated with α-synuclein	Increased opening of Cx43 and Panx1 HCs	Increased release of ATP and oxidative stress	Diaz et al., 2019
	Acute PD animal model induced by MPTP treatment	Increased astroglial Cx43 and Cx30 expressions in striatum	Astrogliosis; astrocyte neuroprotection	Fujita et al., 2018
Multiple sclerosis	EAE mouse model	Increased Cx43 expression in the lumbar spinal cord	Disruption of glial syncytium; inflammation	Takase et al., 2024
	Human *post-mortem* cervical spinal cord tissues from MS patients	Increased Cx43 expression in demyelinated lesions	Dysfunction of astrocyte network	Lieury et al., 2014
	Acute phase in EAE mouse model	Reduced Cx47 and Cx32 GJs around MS lesions	Demyelination and axonal loss	Markoullis et al., 2012a
	Human *post-mortem* brain samples from MS patients	Reduced Cx32 and Cx47, and increased Cx43 GJs levels around MS lesion	Alterations in myelin structure and function	Markoullis et al., 2012b
Huntington’s disease	Human *post-mortem* HD brain samples	Increased Cx43 expression in caudate nucleus	Astrogliosis; alterations in the neuronal environment	Vis et al., 1998

**FIGURE 2:**
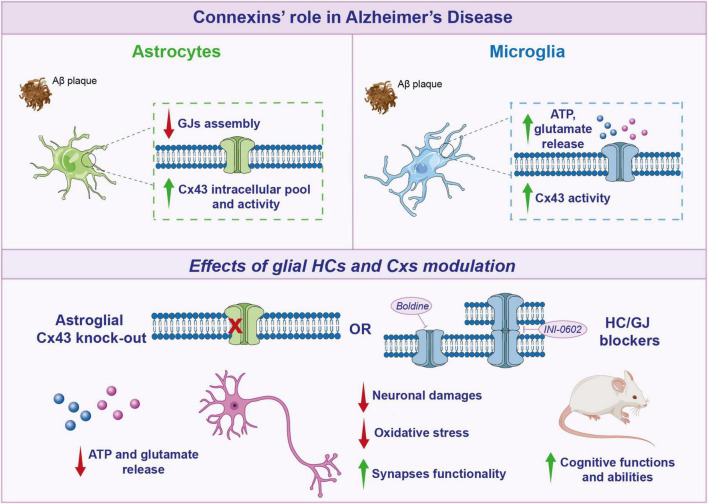
Connexins’ role in Alzheimer’s disease. Schematic representation of glial connexins (Cxs) modulation in Alzheimer’s disease (AD). In astrocytes, Aβ decreases gap junctions (GJs) assembly, but increases Cx43 intracellular pool and activity. After Aβ treatment, also microglia show an increase in Cx43 activity, with a higher ATP and glutamate release. Targeting glial Cxs, through Cx43 knockout (KO) or hemichannels (HCs) blockers, induces a beneficial effect, leading to a reduction in gliotransmitters release, neuronal damage, oxidative stress, and a subsequent improvement in cognitive functions in AD mouse models.

Alterations in glial expression and function of GJs are implicated in AD neurotoxicity (Bosch and Kielian, 2014). *In vitro* studies have shown that Aβ_25–35_ decreased GJs assembly but increased Cx43 intracellular pool and activity, indeed Aβ can alter Cx43 function in astrocytes through an increased internalization (Maulik et al., 2020; Figure 2). Similarly, in APPswe/PS1dE9 mice, a mouse model of familial AD, it has been reported an increased Cxs expression in astrocytes nearby amyloid plaques in murine brain (Yi et al., 2016).

Reactive astrocytes exposed to amyloid plaques showed an increased activity of HCs as compared to the overall astrocyte population (Asghari et al., 2024). Cx43-based HCs were the main factors involved in such a phenomenon, but also pannexin1 (Panx1)-formed channels played a role in reactive astrocytes surrounding amyloid plaques. In particular, Cx43 HCs activity was triggered by enhanced calcium levels into astrocytes, instead Panx1 activation was prompted by inflammation (Gomez et al., 2024).

Such an increased HC and pannexon activity in astrocytes can be associated with toxic effects mediated by small molecules, such as ATP and glutamate, acting on either neurons and/or microglia.

Before the development of Aβ deposits, young APP/PS1 mice at 2 months showed Cx43 and Cx30 distributions superimposable to that of control mice. Instead, in older animals (4 months), Cxs expression was altered nearby dense core of Aβ plaques, while 18 months-old mice presented a high immunoreactivity for both Cxs in most of the plaques (Mei et al., 2010).

In contrast, a model of early AD exhibited an altered HCs activity in hippocampal astrocytes. Mice with intracerebroventricular (icv) injection of Aβ_1–42_ showed increased Cx43 phosphorylation levels, demonstrating that HCs activity is also regulated by AD progression (Madeira et al., 2023).

Indeed, in the early phases of AD, murine models showed apparent discrepancies between 2 months-old APP/PS1 mice versus Aβ_1–42_ icv-injected mice. Such observations can be attributed to the direct increase in Aβ_1–42_ levels in the icv model, as opposed to the gradual accumulation processes characterizing APP/PS1model, where potential compensatory phenomena might take place during the early stages of the disease (Lopez-Gonzalez et al., 2015). As a matter of fact, older 5XFAD mice, a widely used AD mouse model, showed higher expression of Cx43 and Cx30, while Cx47 was reduced (Angeli et al., 2020). Interestingly, the reduction of Cx47 has been linked to impaired metabolic support provided by oligodendrocytes to neurons, leading to a subsequent decrease in myelination (Angeli et al., 2020). Furthermore, reduced colocalization of Cx47 GJs plaques with Cx43 plaques has been observed, suggesting a shift toward the formation of astrocyte-astrocyte GJ and/or HCs rather than astrocyte-oligodendrocyte connections. This shift could further compromise oligodendrocytes homeostasis, contributing to the dysregulation of myelination processes and exacerbating axonal dysfunction (Angeli et al., 2020).

In astroglial targeted-Cx43 knockout (KO) in APP/PS1 mice it has been observed a reduction in neuronal damage and a decreased oxidative stress in neurons localized in hippocampus and related to amyloid plaques, coupled with improved neuronal functionality (Yi et al., 2016). In addition, in APP/PS1 mice, deletion of astroglial Cx43 reduced astrogliosis and increased synapses functionality but did not influence amyloid plaques arrangement. Cx43 KO mice showed decreased levels of factors involved in astroglial activation, such as an increase in GFAP positive cells and GLAST mRNA expression levels. Moreover, astroglial Cx43 KO in APP/PS1 mice enhanced cognitive functions and, re-expression of Cx43 reverted these beneficial effects (Ren et al., 2018).

Besides mice models, *post-mortem* AD brains also showed an increased Cx43 immunoreactivity in astrocytes in contact with amyloid plaques (Nagy et al., 1996). Furthermore, Aβ stimulates IL-1β and TNF production in astrocytes and increases Cx43-based HCs activity. Of note, inhibition of IL-1β and TNF signaling axis reverted this phenomenon (Orellana et al., 2011; Gajardo-Gomez et al., 2017).

The gene encoding Cx43 (Gja1) is the top key driver of an astrocyte enriched subnetwork correlated to AD, regulating the expression of multiple AD risk factor genes (Kajiwara et al., 2018; Zhao et al., 2021). Indeed, Gja1 is upregulated throughout AD progression and is involved in the modulation of inflammatory and immune processes in astrocytes (Boulay et al., 2018). Moreover, an RNA-seq analysis on Gja1^–/–^ astrocytes revealed that Gja1 controls the expression of numerous genes implicated in Aβ metabolism. Indeed, Gja1^–/–^ astrocytes exhibited decreased Apolipoprotein E levels and altered Aβ phagocytosis, revealing a substantial neuroprotective function (Kajiwara et al., 2018; Nanclares et al., 2021).

Increased expression and hyperactivity of Cx43 in astrocytes induces the release of gliotransmitters, such as ATP and glutamate. ATP stimulates astroglial purinergic P2Y1 receptors, causing astrogliosis through autocrine signaling, while the release of glutamate induces excitotoxicity and neuronal death (He et al., 2020). Moreover, many studies confirmed that pro-inflammatory cytokines, such as TNF, could reinforce glutamate release from astrocytes and microglia, leading to neurotoxicity (Takeuchi et al., 2006; Bennett et al., 2012). In addition, another mechanism involved in the pathophysiology of AD could be the enhanced intracellular calcium levels, implicating the activation of endoplasmic reticulum stress, following Cx43 overexpression (He et al., 2020).

An increase in Cx43 and Panx1 activities was also observed in microglia after Aβ_25–35_ treatment. ATP and glutamate released in extracellular medium from microglia treated with Aβ_25–35_ can induce HCs opening in neurons, leading to neurotoxic alterations and harmful effects (Orellana et al., 2012).

Pharmacological blockade of Cxs functionality has shown promising effects in modulating neuroinflammation and cognitive function. INI-0602 is a GJ inhibitor that, by binding to the outer loop of Cxs, leads to the irreversible internalization of Cxs, effectively blocking GJ/HC formations. Indeed, in APP/PS1 mice, it has been demonstrated that INI-0602 was able to inhibit microglia derived glutamate, with a consequent improvement in murine cognitive abilities (Figure 2; Takeuchi et al., 2011). Interestingly, the use of boldine, an alkaloid from the boldo tree that acts by blocking the activity of HCs has shown promising effects, although the mechanism of action is not fully understood (Yi et al., 2017). A long-term boldine administration in APP/PS1 mice reduced glial HCs activity and the consequent ATP and glutamate release, improving neuronal fitness.

Recently, evidence showed that adenosine A2A receptors (A2AR) are involved in the regulation of Aβ effects on Cx43 HCs activity in astrocytes, indeed A2AR pharmacological and genetic blockade prevented HCs alteration related to Aβ. Moreover, throughout primary cultures of astrocytes, it has been demonstrated that protein kinase C (PKC) pathway was involved in these A2AR-induced effects. Thus, A2AR can modulate HCs activity of hippocampal astrocytes in an early AD phase (Madeira et al., 2023). Moreover, A2AR antagonists blocked ATP release mediated by Cx43 HCs in Aβ_1–42_-exposed astrocytes (Madeira et al., 2021). Taken together, this evidence suggests that targeting glial Cxs could be a potential effective strategy to reduce neurotoxicity and ameliorate AD patients’ cognitive functions.

## 3 Connexins’ role in Parkinson’s disease (PD)

**FIGURE 3:**
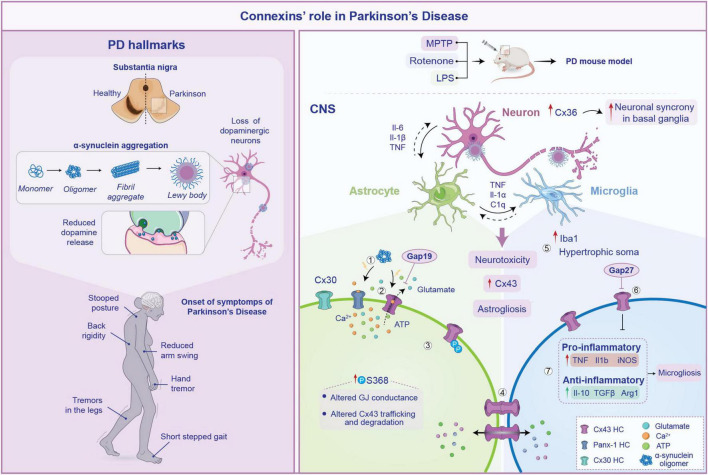
Pathogenesis of Parkinson’s disease (PD) and connexin involvement. Pathophysiology of PD is schematized on the left. Several processes are involved in its pathogenesis including loss of substantia nigra neurons, α-synuclein aggregation from monomers to Lewy bodies and subsequent reduction in dopamine release. These neurodegenerative changes lead to the common PD symptoms such as tremor at rest, stooped posture and gait disturbances. On the right, the role of connexin (Cxs) in the neuroinflammatory response within the central nervous system (CNS) during PD is depicted. Aggregation of α-synuclein triggers Cx43 HCs opening (1), which abnormally release signaling molecules such as ATP and glutamate (2) contributing to neurotoxicity. Furthermore, increased phosphorylation of Cx43 at serine 368 impairs gap junction (GJ) conductance, reducing Cx43 opening and promoting its internalization. This limits the formation of functional GJs, compromising their ability to maintain extracellular homeostasis (3). The increased astrocytes-microglia intercellular communication mediated by Cx43 fuel astrogliosis, facilitating the exchange of pro-inflammatory cytokines (4). In particular, in PD mouse models an increase in Iba1 expression and microglial hypertrophic soma has been observed (5). This is accompanied by heightened release of pro-inflammatory molecules, along with anti-inflammatory cytokines, suggesting a chronic activation of microglia (6). Notably, inhibiting Cx43 HCs with Gap27 reduces this effect, contributing to reduce astrogliosis spread during PD (7).

Parkinson’s disease is a progressive neurodegenerative disease, with a global prevalence of more than six million individuals (Tolosa et al., 2021). PD is characterized by a degeneration of dopaminergic neurons in the substantia nigra pars compacta and the abnormal accumulation of α-synuclein, forming Lewy bodies and Lewy neurites. The disruption of dopaminergic transmission leads to the onset of extrapyramidal motor symptoms, including tremor at rest, bradykinesia and akinesia (Figure 3; Bloem et al., 2021). Moreover, PD is also associated with a variety of non-motor symptoms, such as memory loss, depression, pain and sleep disorders (Tolosa et al., 2021). Although the exact molecular mechanism of PD remains poorly understood, neuroinflammation, oxidative stress and alteration of CNS architecture are considered as key factors in its pathogenesis.

In PD, disruptions in the intricate neuroglia crosstalk have emerged as pivotal contributors to the disease’s pathogenesis and progression. Accumulating evidence suggest an active role for non-neuronal cells, where the possible alteration of astrocytes and microglial functions and the disruption of neuron-to-glia crosstalk may contribute to PD pathogenesis and progression (Guo et al., 2020; Kam et al., 2020). Aberrant functioning of Cxs-based channels and related signaling pathways in glial networks has been implicated in dopaminergic neuronal degeneration, neuroinflammatory responses, and impaired neuroprotection (Kawasaki et al., 2009; Saez et al., 2010; Table 1). Thus, the molecular signaling mediated by glial GJs appears to be critical in this context. The maintenance of the physiological concentration of several molecules at cytoplasmic and extracellular levels, such as glutamate and ions like potassium, as well as the regulation of pH in astrocytes, largely depends by Cx43 functionality (Vicario et al., 2020). These mechanisms are of critical importance in the regulation of dopaminergic neurons firing and homeostasis (Wang et al., 2020). This suggests that changes in the levels of Cx43 HCs or alterations in the Cx43 gating mechanism are key processes in the development of the disease.

Over the last decades, numerous studies have focused their attention on alteration of Cx43 dynamics and its role in the progression of PD. Kawasaki and collaborators showed an increase of Cx43 expression in rotenone-induced PD model, both *in vitro* and *in vivo* (Kawasaki et al., 2009). Rotenone is a mitochondrial complex I inhibitor, widely used to induce oxidative stress and subsequent cell death, mimicking parkinsonism in rodents when injected systemically (Betarbet et al., 2000). Increased protein levels of Cx43 were detected in the substantia nigra pars compacta and pars reticulata, and striatum, suggesting that aberrant coupling between glial cells could be crucial in the neurodegenerative process that drive PD development (Kawasaki et al., 2009). In addition, rotenone treatment promoted the phosphorylation of Cx43, which is critical for intercellular communication properties occurring at several stages of the Cx “life cycle,” including trafficking and degradation, assembly/disassembly and permeability (Kawasaki et al., 2009; Zhang et al., 2023). However, other evidence reports a marked reduction in Cx43 expression and permeability on rotenone-stimulated primary cultured astrocytes, which is associated with impaired GJs communication and increased apoptosis (Zhang et al., 2011). Such a discrepancy could be related to the different concentration of rotenone used in these studies. While the former used a concentration of 8 nM rotenone, the latter used a concentration in the range of 20–30 nM. This could suggest that at lower doses, rotenone may enhance the phosphorylation of Cx43, potentially increasing gap junctional intercellular communication, while higher concentrations may induce a different effect by disrupting Cx43 functionality.

Besides its direct effects on astrocytic functions and survival, Cx43-mediated signaling has been shown to promote neuroinflammatory response following CNS insults (Denaro et al., 2024). Crosstalk between activated microglia and astrocytes is thought to be a key regulator of neuroinflammation in PD (Grotemeyer et al., 2022). Via immunofluorescence staining, an increase in Cx43 intensity was reported in the substantia nigra of animals that received brain injections of lipopolysaccharide (LPS) (Voronkov et al., 2023). LPS has the capability to induce inflammatory-driven dopaminergic neurons degeneration and has been used as the common toxicant for PD modeling (Gao et al., 2003). Along with this finding, the study showed severe astrogliosis, that was coupled by long-term (up to 3 months) alteration of the glial network with a redistribution of Cx43 in the astrocytes end-feet (Voronkov et al., 2023). This was accompanied by an increase of Iba1^+^ cells (i.e., microglia) with hypertrophic soma around the injection area (Voronkov et al., 2023). It can be speculated that the observed changes in Cx43 localization contributes to the prolonged maintenance of the neuroinflammatory state. One possible hypothesis is that the Cx43 redistribution enhances coupling with microglia, promoting the exchange of pro-inflammatory cytokines within the glial network, thereby sustaining the reactive gliosis. Indeed, an increase of mRNA expression levels of typical pro-inflammatory markers (TNF, Il-1β and iNOS) has been observed, with an increase in the levels of the anti-inflammatory cytokines (Il-10, TGFβ and Arg1) in a model of LPS-induced PD, suggesting a chronic activation of microglia (Zhao et al., 2022). Interestingly, the injection of Gap27, a mimetic peptide able to selectively block Cx43-based GJs and HCs, mostly attenuated microgliosis, leading to a decrease of the inflammatory response triggered by LPS. Moreover, Cx43 inhibition was able to attenuate the damage to dopaminergic neurons as well as the reduction of dopamine and its metabolites *in vivo* caused by LPS (Zhao et al., 2022; Figure 3).

Furthermore, it has been demonstrated that α-synuclein might affect astrocytic HC function, triggering the opening of Cx43- and Panx1-based channels, leading to abnormal calcium signaling, excessive release of gliotransmitters such as ATP and glutamate and increased oxidative stress. Consistent with this evidence, knockdown of Cx43 with siRNA or blocking with Gap19, completely abolished ATP release induced by α-synuclein (Diaz et al., 2019). These changes contribute to a vicious cycle of astrocytic dysfunction and neuronal degeneration, which fuel neuroinflammation (Figure 3).

In addition, studies have shown that α-synuclein oligomers can bind to Cx32, facilitating their uptake by neurons and oligodendrocytes (Reyes et al., 2019). Evidence from *post-mortem* brain tissue of PD patients further supports a direct interaction between α-synuclein and Cx32. This interaction promotes the intercellular transfer of α-synuclein, thereby contributing to the propagation of PD (Reyes et al., 2019).

Another important piece of evidence highlighting the contribution of GJs coupling in PD comes from a study conducted by Schwab and colleagues, showing the critical role of the neuronal Cx36 in enhancing neuronal synchrony in the basal ganglia. This study, conducted on *post-mortem* human tissues, revealed a significant overexpression of Cx36 in putamen and globus pallidus (GP) of PD patients, highlighting its role in pathological network activity associated with the motor deficits observed in PD (Schwab et al., 2014).

Modulating Cxs expression and gating properties could be a key strategy in understanding the pathogenesis of PD, by interfering with the inflammatory cascade (Green and Nicholson, 2008). To this end, the effects of conditional inactivation of the glucocorticoid receptor (GR) on astrocytes were investigated in GR^*Cx*30*CreERT*^ mice, using a model of PD induced by 1-methyl-4-phenyl-1,2,3,6-tetrahydropyridine (MPTP) injection (Maatouk et al., 2019). This study found that the deletion of GR on astrocytes led to a significant loss of dopaminergic neurons after MPTP treatment in the substantia nigra of mutant mice. Furthermore, in the absence of GR, it has been observed an increased activity of Cx43 HCs that, not only enhanced calcium levels in astrocytes, but also trigger microglial reactivity following MPTP induction, causing a robust inflammatory response. This was coupled with increased levels of specific inflammatory markers, including intercellular adhesion molecule 1 (ICAM-1), TNF and Il-1β. Analyses of *post-mortem* brain tissue from PD patients revealed a significant decrease in the number of astrocytes expressing GR in the substantia nigra, suggesting the existence of a link between the neuroinflammatory cascade and the activity of Cx43 (Maatouk et al., 2019).

Astrocytes, through GJs, sculpt an environment that ensures proper neuronal functions. In addition to Cx43, astrocytes also abundantly express Cx30, which is mainly located in astrocytic processes around neurons in the gray matter, constituting an important component of the astrocytic metabolic network (Nagy et al., 1999). It has been observed that both Cx43 and Cx30 are upregulated in astrocytes in the striatum of an acute PD animal model induced by MPTP treatment (Fujita et al., 2018). However, Cx30 KO mice showed an increased susceptibility to MPTP, exacerbating the loss of dopaminergic neurons. This may suggest that other aspects of Cx functions are required for neuronal survival. Cx30 KO mice, treated with MPTP, showed a reduced expression of genes associated with the neuroprotective response of astrocytes in the striatum as compared to wild-type (WT) mice (Fujita et al., 2018). This may suggest that Cx30 is involved in the regulation of the neuroprotective properties of astrocytes within the tissue; thus, enhancing Cx30 function could be a potential therapeutic approach for PD patients.

## 4 Connexins’ role in multiple sclerosis (MS)

Multiple sclerosis (MS) is a chronic, autoimmune, inflammatory disorder of the CNS characterized by progressive demyelination, neuroinflammation, and neurodegeneration (Orton et al., 2006). A number of factors associated with the onset of MS have been reported to be related to the incidence of the disease. For example, an increase in MS prevalence is observed with the distance from the equator and in certain geographical areas (Compston and Coles, 2008). Relapsing remitting MS (RRMS) is the most common clinical form of MS affecting 85–90% of people aged 20–40 years old and it is characterized by symptomatic attacks (relapses) followed by periods of recovery (remissions). RRMS frequently develops into secondary progressive MS (SPMS), characterized by the progressive and irreversible accumulation of neurological impairment (Hauser and Cree, 2020). The involvement of Cxs-based intercellular communication in MS has garnered significant attention due to their role in glial cell, inflammation, and myelination. Redistribution of Cxs plaques and modulation of intercellular exchanges has been linked to either sustained inflammation and defects in myelination, highlighting the need of further research into Cxs influence on immune response and remyelination (Table 1).

**FIGURE 4:**
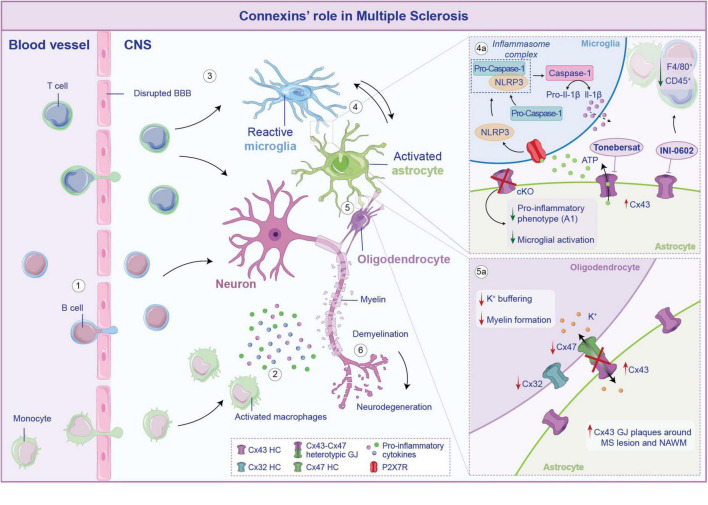
Connexins (Cxs)-mediated neuroglia crosstalk in multiple sclerosis. In multiple sclerosis (MS) the blood-brain barrier (BBB) becomes compromised, allowing B cells, T cells and monocytes to infiltrate the central nervous system (CNS) (1). Once crossed the BBB, activated macrophages secrete pro-inflammatory molecules that exacerbate neuronal suffering (2). The infiltrating immune cells trigger the activation of microglia (3) that, in turn, stimulate astrocytes toward a pro-inflammatory phenotype, establishing a self-perpetuating cycle of inflammation (4). Cx43-mediated crosstalk between astrocytes and microglia further elicited these processes. Specifically, Cx43 opening leads to ATP release that, acting on P2X7R on microglia, activates the NLRP3 inflammasome complex, leading to the cleavage of pro-caspase-1 into active caspase-1, which then process pro-Il-1β into its mature forms, released in the cytosol (4a). Conditional knockout (cKO) on astrocytes or blockade of Cx43 HCs leads to a reduction of microglial activation and a downregulation of genes associated with the pro-inflammatory A1 phenotype in astrocytes. Cx43 is also involved in the crosstalk between astrocytes and oligodendrocytes (5). If from the one hand an increase of Cx43 has been observed during MS, on the other hand the neuroinflammatory milieu leads to a reshaping of the overall glial network, which comprises the downregulation of oligodendrocytic Cx32 and Cx47 (5a). Disruption of heterotypic Cx43-C47 GJ affects potassium buffering and myelin formation, ultimately contributing to neuronal demyelination and neurodegeneration (6).

Multiple sclerosis is the result of a primary defect in the immune system, which mediates direct damage to the oligodendrocytes, the CNS myelinating cells, resulting in secondary damage to neurons. This aberrant environment leads to the activation of the CNS-resident cells such as microglia and astrocytes. To summarize, from a neuropathological point of view, MS hallmarks include lesion in the CNS white and gray matter areas with variable degree of demyelination, immune cells infiltration, reactive gliosis and neurodegeneration (Figure 4; Compston and Coles, 2008; Charabati et al., 2023).

In this scenario, Cxs-mediated neuroimmune crosstalk undoubtedly plays an active role in driving the neurodegeneration process and it is increasingly recognized as a critical factor in disease progression (Charabati et al., 2023). However, the exact mechanism by which Cxs activity influences these processes remains controversial. Evidence of altered Cxs protein has been observed in MS patient biopsies as well as in murine preclinical model. Notably, increased expression of Cx43 has been reported in the experimental autoimmune encephalomyelitis (EAE) model of MS. EAE is a well-established and widely used preclinical model, where animals are immunized with myelin-derived peptides, resulting in a progressive disease resembling the relapsing-remitting or progressive forms of MS (Constantinescu et al., 2011). Takase et al. (2024) reported changes in Cx43, observing an overexpression in the lumbar spinal cord of EAE mice throughout the time course of the disease. Evidence from human *post-mortem* cervical spinal cord tissues confirmed an upregulation of Cx43 in peri-plaque demyelinated lesions, coupled with an accumulation of macrophages and microglia (Lieury et al., 2014).

In accordance with its role in sustaining MS progression, recent reports indicate that astroglia-specific Cx43 ablation attenuates EAE clinical signs, with reduced F4/80^+^ macrophages and CD45^+^ cells infiltration (Une et al., 2021).

Additionally, administration of INI-0602, a pan-Cx blocker that inhibits GJ/HC opening (Umebayashi et al., 2014) demonstrated a therapeutic effect on EAE mice, by preventing excessive demyelination in the spinal cord, as evidenced by an increase of the myelin basic protein-immunopositive (MBP^+^) area in INI-0602-treated mice. The effects of Cx43 deletion/blockade are also evident in the regulation of the neuroinflammatory environment during the course of EAE. Astroglia-Cx43 conditional (c-)KO mice showed reduced activation of astrocytes toward a pro-inflammatory (A1) phenotype during acute EAE, and enhanced anti-inflammatory responses in spinal microglia before the onset of the EAE. However, during the peak phase of the disease, both wild-type (WT) and Cx43cKO mice displayed similar pro-inflammatory profiles (Une et al., 2021).

*In vivo* assessment further showed that the blockade of Cx43 HCs by INI-0602, suppressed microglial activation, as evidenced by a more ramified morphology, and decreased astrocytes activation in the spinal cord. Additionally, INI-0602 administration decreased levels of pro-inflammatory cytokines (Il-6, INF-γ) and increased anti-inflammatory Il-10 in the cerebrospinal fluid (CSF) of EAE mice. These findings suggest that activated microglia drive astrocytes toward a pro-inflammatory phenotype, exacerbating neuroinflammation (Takase et al., 2024).

One proposed mechanism by which Cx43 exacerbates and sustains the neuroinflammation in MS is linked to the aberrant release of ATP, which serves as activation signal of the NOD-like receptor protein 3 (NLRP3) inflammasome (Malhotra et al., 2020; Kwakowsky et al., 2023). The blockade of Cx43 HCs, mediated by tonebersat in EAE model, effectively reduces astrocytes and microglia activation, thus slowing disease progression. This, in turn, limits the formation of the NLRP3 complex that correlates with lower levels of Caspase-1 cleavage, which are crucial steps in inflammasome-driven neuroinflammation (Figure 4; Kwakowsky et al., 2023).

In addition to direct ATP release, Cx43 HCs may facilitate the propagation of the neuroinflammatory response acting downstream to the P2X7 receptor (P2X7R)-Cx43-β_3_ integrin axis. This receptor is activated by ATP released from astrocytes through different mechanisms, including Cx43 HCs. Analyzing spinal cord of EAE rats, Milicevic et al. (2023) found a close interaction between these two proteins, particularly near infiltrated CD4^+^ T cells. Microglial cells also express the P2X7R, thus it is plausible that a similar mechanism of interaction occurs in microglia, thereby contributing to neuroinflammation. Furthermore, this study highlighted the role of mitochondria in regulating astrocytic coupling. Blocking specific mitochondrial transporters significantly augmented the ATP release from astrocytes, enhancing the immune cell-induced astrocytic calcium response (Milicevic et al., 2023). Given that Cx43 is not only localized at the plasma membrane, but also within mitochondria, another layer of complexity must be considered (Boengler et al., 2022). This means that the mitochondrial localization of Cx43 may have a direct influence on astrocytic mitochondrial functions, including the regulation of calcium dynamics in response to cellular stress.

Thus far, if from the one hand there is the increasing assumption that high levels of Cx43 are detected in the CNS during EAE progression, on the other hand, different lines of evidence reveal a more nuanced and controversial role of Cx43. It has been observed that mutant mice lacking both Cx43 and Cx30 exhibited an altered neurochemical microenvironment and hypomyelinating phenotype (Lutz et al., 2009). However, these changes did not translate into more severe EAE clinical symptoms or increased inflammatory cells infiltration (Lutz et al., 2012).

The idea that GJs, particularly Cx43 in astrocytes, may have distinct roles at different stages of EAE is increasingly convincing. Indeed, the study of Lutz and colleagues takes into account a narrow window of time, between the onset and the peak of the disease (i.e., 25 days post immunization, dpi). It is possible that early microenvironmental changes caused by the deletion of Cx43 could alter myelination without directly influencing the severity of clinical symptoms, which may depend more on immune cells infiltration and neurodegeneration, taking place in the chronic phase (Lutz et al., 2012). This is further supported by the observation that pharmacological blockade of Cx43 resulted in a significant improvement in EAE clinical score, particularly evident around 22 dpi and became more consistent at 50 dpi, during the relapsing phase (Une et al., 2021). Another important consideration is the possibility that, although Cx43 is a key player in forming GJs, other Cxs may partially compensate for its absence, ensuring some level of communication between glial cells.

Oligodendrocytes express both Cx32 and Cx47, which form GJs with their main astrocytic partner Cx43 (Kamasawa et al., 2005). The homomeric combination of Cx43-Cx47 is critical for maintaining potassium buffering, nutrient homeostasis and myelin formation properties by oligodendrocytes (Basu and Sarma, 2018).

A redistribution of Cx47-formed GJs plaque to the intracellular compartment has been observed following the loss of Cx43, at 14 dpi subsequent EAE induction (Markoullis et al., 2012a). In line with this evidence, a similar distribution pattern was found on human *post-mortem* brain samples from MS patients, that revealed reduced Cx32 and Cx47, and increased Cx43 GJ plaques around demyelinating lesions and on the normal appearing white matter (NAWM) (Figure 4). Moreover, there was an increase of Cx47 in oligodendrocytes precursors cells (OPCs), which are recruited to areas of demyelination. However, these OPCs exhibited limited coupling with astrocytes as indicated by the reduced formation of heterotypic Cx43–Cx47 astrocyte-to-oligodendrocyte GJs (Markoullis et al., 2012b; Masaki et al., 2013). Cx47 has been studied in the context of myelinogenesis and demyelination and has been associated with developing oligodendrocytes and in specific CNS areas in the adulthood (Parenti et al., 2010). It is interesting that Cx47 expression in oligodendrocytes is an early effect anticipating Cx32 and Cx29 expression, all typical Cxs expressed by mature oligodendrocytes (Vicario et al., 2017). It has been demonstrated that Cx47 is highly expressed by astrocytes during demyelination and that a shift in Cx47 expression from astrocytes to oligodendrocytes is taking place during spontaneous remyelination. Thus, sustaining Cx47-based intercellular crosstalk between astrocytes and oligodendrocytes might be of critical importance during remyelination (Parenti et al., 2010).

To summarize, the common idea that comes up from the above-mentioned mechanism is that an imbalance in Cxs expression during MS may result in the formation of unpaired HCs, disrupting glial syncytium and promoting inflammation and demyelination.

## 5 Connexins’ role in Huntington’s disease (HD)

Huntington’s disease is a neurodegenerative disorder, characterized by abnormal long CAG repeat expansions in the Huntingtin gene, that causes the expansion of a polyglutamine (polyQ) stretch in the huntingtin protein (HTT) (Shin et al., 2005; Mielcarek et al., 2014). The mutant huntingtin (mHTT) shows altered functions and protein-protein interactions, leading to neurological symptoms and brain pathology (Mielcarek et al., 2014). Indeed, HTT is expressed in different tissues and, intracellularly, colocalizes with many organelles: nucleus, Golgi apparatus and endoplasmic reticulum (Strong et al., 1993; Imarisio et al., 2008). HTT plays important roles in different pathways due to its scaffolding role in protein complex formations, indeed more than 200 interaction partners are known and studied (Alteen et al., 2023).

Intercellular communication is vital for maintaining neuronal homeostasis and supporting physiological neuronal function. The HD early phase is characterized by mutant HTT self-aggregation, involved in the appearance of pathological symptoms (Gutekunst et al., 1999; Marquette et al., 2021). Moreover, mHTT leads to a progressive neuronal dysfunction and neuronal death in the striatum and cortex, resulting in motor dysfunction and cognitive impairment (Ross et al., 2014). Neuroinflammation is correlated with HD pathophysiology, indeed reactive astrocytes and microglia are detectable in brain regions involved in HD (Sapp et al., 2001; Rocha et al., 2021). Reactive astrocytes and microglia, expressing mutant HTT, play central roles in neuroinflammation, synaptic dysfunction, and neuronal death. Altered Cxs-mediated signaling, impaired glial homeostasis, and changes in myelinogenic potential further exacerbate the pathophysiological cascade in HD (Palpagama et al., 2019; Giaume et al., 2021; Table 1).

At the beginning, inflammatory mechanisms are effective to compensate the alterations related to protein aggregations and deposits; however, inflammation can cause neuronal impairment, supporting HD degeneration. Several *post-mortem* studies revealed a correlation between HD severity and the levels of astroglial and microglial activation (Rocha et al., 2021). Furthermore, mHTT can induce the activation of microglia, that undergo morphological changes, hyperproliferation and production of inflammatory cytokines. These cytokines could alter microglia phagocytic capacity, leading to mHTT accumulation and its consequent detrimental effect (Michelucci et al., 2009; Palpagama et al., 2023). Moreover, microglia could also play a significant role in synaptic impairment during HD progression (Savage et al., 2020).

Recently, it has been demonstrated that microglia from WT mice in co-culture with mHTT striatal neurons had an amoeboid shape, an augmented proliferation and cytokine IL-6 levels, and, despite their activation, they increased mHTT neuronal viability (Savage et al., 2020). Conversely, mHTT microglia seems to induces neuronal suffering and cell death, *in vivo* (Crotti et al., 2014). Reactive microglia and altered cytokine levels were also detected in the brains of HD patients (Crotti et al., 2014).

Similarly, from the analysis of *post-mortem* HD brains, it has been observed that also astrocytes express mHTT. In co-cultures, mHTT astrocytes lead to striatal neurons death, while in a double transgenic model, mHTT astrocytic expression significantly altered neuronal functions (Bradford et al., 2010; Diaz-Castro et al., 2019; Khakh and Goldman, 2023). Using a snRNAseq, it has been demonstrated that astrocytes in *post-mortem* human HD cingulate cortex showed multiple expression profiles, that could be grouped in three states with different levels of GFAP, metallothionein and quiescent protoplasmic genes expression levels. Moreover, as compared to control, HD astrocytes exhibited a downregulation of genes involved in protoplasmic astrocyte function and lipid synthesis (Al-Dalahmah et al., 2020).

Pathological changes were also observed in microglia and astrocytes localized in the midcingulate cortex, a brain area involved in motor and cognitive functions. In particular, these alterations were observed in HD cases with prevalent mood symptoms (Palpagama et al., 2023).

Recently, some studies revealed that HD patients presented demyelination and white matter loss, that could originate before symptoms (Tabrizi et al., 2012). Thus, an alteration in myelinogenic oligodendrocytes may be related to HD pathophysiology (Teo et al., 2016). Osipovitch et al. (2019) demonstrated that glial progenitor cells derived from human embryonic stem cells (hESCs) expressing mHTT show an altered maturation, leading to dysfunctional astrocytes and demyelination *in vivo*. In addition, mHTT human glial chimeras show an impaired astrocyte differentiation, confirming that defects in glial differentiation could be related to HD pathogenesis and symptoms (Osipovitch et al., 2019).

Benraiss et al. (2016) engrafting mice with mHTT-expressing human glial progenitor cells, revealed that mHTT glia can induce HD phenotype in healthy mice, also showing a decline in motor performance. However, normal glia can improve behavior and survival of transgenic HD mice, reestablishing interstitial potassium homeostasis and reducing HD progression (Benraiss et al., 2016). These results confirm the important role of glia in HD progression, revealing cell-based treatment as a potential strategy against HD.

In HD human brain, caudate nucleus (CN) and GP of the basal ganglia are areas strongly affected. Examining Cxs expression and distribution in these regions, it was demonstrated that Cx50 was not expressed, while Cx40 was identified in endothelial cells of blood vessels. Instead, Cx26 and Cx32 showed a superimposable distribution both in normal and HD brain, with a higher expression in GP as compared to CN. Cx43 was the only Cx that revealed a significantly different expression between healthy and HD brains. Indeed, Cx43 was increased in CN of HD human brains, and this was correlated with a higher expression of GFAP, that demonstrated a robust astrogliosis (Vis et al., 1998; Huang et al., 2021). However, Cxs role in HD has not been fully elucidated and new studies about this topic are needed.

## 6 Conclusion

In recent years, several efforts have been made to broaden the knowledge of the processes that drive the progression of neurodegenerative diseases. This review highlighted the role of Cxs in glial communication, neuroinflammation and the disruption of homeostasis in neuroinflammatory-driven diseases, which are summarized in Table 1.

All explored neurodegenerative disorders, AD, PD, MS and HD, appear to be coupled with a general overexpression of Cx43 and increased intercellular communication between astrocyte and microglia.

This set of evidence supports the idea that Cxs upregulation, particularly Cx43, is a critical and a common factor in neurodegenerative diseases, where its act as “pathological” pore, supporting the notion that Cxs are not merely passive markers of disease, but active contributors to its progression. This makes Cxs ideal targets for therapeutic strategies aimed at reducing inflammation and restoring cellular homeostasis.

However, the complexity in their regulation must be considered. For instance, phosphorylation state of Cx43, its interaction with other cellular proteins, and its localization within cells and mitochondria can all impact its role in disease. Last but not least, the non-channel functions of Cxs are of particular importance, such as the ability to bind to DNA and RNA, exerting a modulatory function of genes with consequent functional impact (Kotini et al., 2018). This means that the ideal therapeutic strategy should target specific aspect of Cxs function, without disrupting their beneficial roles in maintaining homeostasis.
